# Utilization of Cryopreserved Oocytes in Patients With Poor Ovarian Response After Planned Oocyte Cryopreservation

**DOI:** 10.1001/jamanetworkopen.2023.49722

**Published:** 2024-01-02

**Authors:** Yuval Fouks, Denny Sakkas, Pietro E. Bortoletto, Alan S. Penzias, Emily A. Seidler, Denis A. Vaughan

**Affiliations:** 1 Boston IVF-The Eugin Group, Waltham, Massachusetts; 2 Harvard T.H. Chan School of Public Health, Boston, Massachusetts; 3 Department of Obstetrics and Gynecology, Beth Israel Deaconess Medical Center, Boston, Massachusetts; 4 Department of Obstetrics, Gynecology and Reproductive Biology, Harvard Medical School, Boston, Massachusetts; 5 The Faculty of Medicine Tel Aviv University, Tel Aviv, Israel

## Abstract

**Importance:**

Poor ovarian response (POR) to stimulation may impact patients’ desire or need to utilize cryopreserved oocytes for family building in the future. These findings, captured by Society for Assisted Reproductive Technology (SART) national data, underscore the need for tailored counseling and further research into the decision-making processes influencing oocyte utilization.

**Objective:**

To examine the association of ovarian response to stimulation and the number of vitrified oocytes with the likelihood and timing of patients returning for oocyte utilization following planned oocyte cryopreservation (OC).

**Design, Setting, and Participants:**

This cohort study used data in the SART Clinical Outcome Reporting System for patients in US fertility clinics and data was used for eligible patients who underwent planned OC from January 2014 through December 2020. Data were analyzed from November 2022 to June 2023.

**Main outcomes and measures:**

The association between number of oocytes cryopreserved on return rate to utilize cryopreserved oocytes and the time from vitrification to warming.

**Results:**

A total of 67 893 autologous oocyte freezing cycles were performed in the US between 2014 and 2020, among 47 363 patients (mean [SD] age, 34.5 [4.7] years). Of these, 6421 (13.5%) were classified as patients with POR, with fewer than 5 oocytes vitrified across all ovarian stimulation cycles. A total of 1203 patients (2.5%) returned for oocyte warming and utilization. The rate of return was significantly higher in the POR group, with 260 (4.0%) returning compared with 943 (2.3%) in the normal responder group (*P* < .001). This trend was most notable in the age 30 to 34 years (warm cycle, 46 of 275 [16.7%] vs no warm cycle, 982 of 11 743 [8.4%]; *P* < .001) and age 35 to 39 years groups (warm cycle, 124 of 587 [21.1%] vs no warm cycle, 3433 of 23 012 [14.9%]; *P* < .001). The time elapsed from vitrification to warming was comparable between patients with POR (mean [SD], 716.1 [156.1] days) and normal responders (803.8 [160.7] days). A multivariate analysis adjusted for age, clinic region in the US, body mass index, and history of endometriosis was conducted to identify factors associated with the utilization of oocytes. The analysis revealed that having fewer than 5 oocytes vitrified was associated with higher odds of utilizing oocytes (OR, 1.52; 95% CI, 1.32-1.76).

**Conclusions and Relevance:**

This cohort study reveals a distinct pattern in the utilization of cryopreserved oocytes among patients undergoing planned OC in the US. Despite the increase in number of patients pursuing OC, there is a notably low rate of return to utilize previously vitrified oocytes; notably, patients with POR are more likely to return, although the time to return is similar to those with normal ovarian response.

## Key Points

**Question:**

Is the amount of cryopreserved oocytes associated with the likelihood and timing of patients returning for oocyte utilization after planned oocyte cryopreservation?

**Findings:**

In this cohort study of 67 893 freezing cycles among 47 363 patients undergoing oocyte cryopreservation, only 2.5% to 3.0% of patients returned to utilize their cryopreserved oocytes following planned oocyte cryopreservation. Patients with poor ovarian response were more likely to return for utilization, although their timing to do so was similar to that of normal responders; factors such as the total number of cryopreserved oocytes, body mass index, and the clinic’s geographic location were significantly associated with patients’ decisions to return to utilize their cryopreserved oocytes.

**Meaning:**

These results suggest that patients with a poor ovarian response are more likely to use their previously cryopreserved oocytes compared with patients with normal ovarian response.

## Teaser

This cohort study of US patients undergoing oocyte cryopreservation examines the association of the amount of oocytes preserved and their subsequent utilization.

## Introduction

Over the past 2 decades, there has been a gradual societal shift toward delaying childbirth.^1,2,3,4^ This, coupled with advancements in technology, has led to a substantial increase in oocyte cryopreservation among patients of reproductive age.^5,6,7^ This process, commonly referred to as elective oocyte cryopreservation and more recently addressed by the American Society for Reproductive Medicine (ASRM) as planned oocyte cryopreservation (OC), offers the potential to mitigate age-related subfertility. However, the lack of follow-up data and limited information on unassisted pregnancy outcomes hinder the ability to draw definitive conclusions regarding its effectiveness.^3,8,9,10,11^

Importantly, while undergoing planned OC, not all individuals experience a response to ovarian stimulation that aligns with their prospective family planning, be it for a desired family size or even the birth of a single child. This indicates that some may not attain the necessary oocyte quality and quantity pivotal for their family building aspirations. These individuals are labeled as “poor responders,” a term that lacks a clear definition.^12,13^ Most of the existing research on patients with poor ovarian response (POR) focuses on those with a concurrent infertility diagnosis.^12,13^

Clinicians are confronted with a challenging clinical dilemma and a paucity of data when counseling patients with POR who have undergone planned OC, given the inherent difference when compared with an infertile patient with POR during conventional assisted reproductive technology (ART) treatment. This is largely due to the relatively low rate of patients returning to utilize their cryopreserved gametes.^8,14^ Increasing the number of planned OC cycles may improve crude live birth rates, but for otherwise fertile patients, this intervention could be time-consuming and costly.^15^ Moreover, because these patients lack a diagnosis of infertility, it is possible that their ovarian response to stimulation, quantitatively, is not associated with reduced future fertility. In many cases, withdrawing from planned OC treatment and attempting to conceive or pursuing ART treatment may be recommended.

The objective of this study is to examine the association between ovarian response during stimulation, as reflected by the quantity of cryopreserved oocytes, and the likelihood and timing of patients choosing to utilize previously cryopreserved oocytes in the context of planned OC. We were particularly interested in investigating the association between POR and the rates of patients returning to utilize cryopreserved oocytes, as observed in a large, national database.

## Methods

Data for this study was obtained from the Society for Assisted Reproductive Technology Clinic Outcome Reporting System (SART CORS). SART is an independent organization that promotes the practice of ART and represents over 85% of US fertility clinics’ annual reporting.^16^ The study period spanned from January 2014 to December 2020. The data were collected through voluntary submission and validated by SART to ensure compliance with the Fertility Clinic Success Rate and Certification Act of 1992.

Institutional review board approval was obtained from the Committee on Clinical Investigations at Beth Israel Deaconess Medical Center. All data received from SART was deidentified. This study followed the Strengthening the Reporting of Observational Studies in Epidemiology (STROBE) reporting guideline for observational studies.

### Study Population

**Figure 1.**
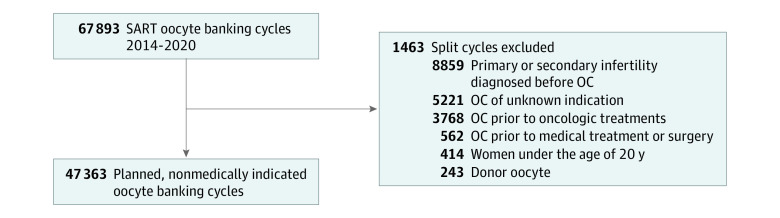
Study Flowchart This flowchart illustrates the patient selection process for planned, nonmedically indicated oocyte cryopreservation (OC). Starting with the total reported OC cycles in the US, we applied multiple exclusion criteria to approximate the base population of our interest—individuals opting for elective OC without prior infertility diagnoses or medical conditions necessitating oocyte preservation. The excluded indications, leading to a final cohort of 47 655 women for analysis. SART indicates Society for Assisted Reproductive Technology.

In our investigation, we narrowed our focus to a specific group: individuals undergoing planned OC for nonmedical, non–infertility-related reasons. To ensure our study cohort reflected this group, we applied criteria to exclude patients with any other indications for oocyte cryopreservation (Figure 1). Our rationale was to isolate an indication and treatment-naive population: those choosing to preserve their fertility for future potential use rather than as a response to a current medical or fertility challenge. We compiled data including age, body mass index (BMI; calculated as weight in kilograms divided by height in meters squared), race, ethnicity, geographic location, the presence of a partner at the time of OC, and the specific indication for egg freezing. Notably, we excluded oocyte donation cycles to maintain focus on those preserving their own oocytes.

An additional layer of specificity in our methodology was the exclusion of cycles in which patients returned for utilization of their cryopreserved oocytes within 60 days after cryopreservation. The intent behind this exclusion was to discount the immediate returns that could be attributed to poor cycle performance and may be diverted into in vitro fertilization (IVF) rather than OC, thereby obtaining a clearer understanding of the long-term utilization patterns of cryopreserved oocytes in our defined population.

In this study, the identification of participant race and ethnicity was conducted using the data reported by the clinics to SART. The source of these classifications was primarily based on the information contained within the clinics’ electronic medical records (EMR). The EMRs typically allow patients to self-report their race and ethnicity, following the standard classifications used in health care settings.

We linked oocyte cryopreservation cycles to their respective thaw cycles using SART data. Our method involved separating unique identifiers for each thaw cycle, which could correspond to multiple egg retrievals, and then systematically matching these thaw cycles with their associated egg freeze cycles. This approach enabled us to accurately track and analyze the utilization patterns of cryopreserved oocytes. Furthermore, identifiers could be linked to several egg retrievals.

### Outcomes

The primary focus of this study was to assess the return rate to utilize cryopreserved oocytes following planned OC, as well as the time to return, in relation to the number of oocytes vitrified. We hypothesized that patients with POR would be more likely to utilize their cryopreserved oocytes earlier compared with those with a normal ovarian response. Additionally, we aimed to examine the association of newly diagnosed POR (patients had opted for planned OC without any prior medical or fertility indications) with the likelihood of utilizing the cryopreserved oocytes.

The initial objective was to analyze the patterns of patient return for oocyte warming and the time interval between freezing and oocyte utilization in planned OC cycles (cryopreservation warm interval). To identify the threshold for POR, we conducted a distributional analysis to determine a cutoff of fewer than 5 oocytes across all cycles. This represents the 25th percentile for oocyte yield across all planned OC cycles of an individual.

To calculate the cryopreservation warm interval, we measured the time elapsed between oocyte cryopreservation and the subsequent warming. This time frame was defined as the time to return. In cases where a patient returned for oocyte warming more than once, we considered the shortest time to utilization, which corresponded to the first medical encounter after cryopreservation, as the reference.

### Statistical Analysis

We employed logistic regression to model and adjust for covariates when evaluating the likelihood of patients returning to utilize their cryopreserved oocytes. Factors such as the total number of cryopreserved oocytes, age at freezing, clinic region in the US, body mass index (BMI; calculated as weight in kilograms divided by height in meters squared), and the presence of endometriosis were included as covariates, selected based on existing fertility research for their potential influence on patient decisions and outcomes.

In addition to the logistic regression, the Cox proportional hazards model was used, after confirming its underlying assumptions, to explore associations between various covariates and the time until patients returned to utilize their cryopreserved oocytes. This model facilitated an analysis of time-to-event data specifically focusing on the timing of patients’ return postcryopreservation. Employing both logistic regression and Cox proportional hazards models allowed for a comprehensive analysis encompassing both the occurrence and timing of oocyte utilization across different regions.

The SART data set showed that 40.6% of the ethnicity data was missing. To maintain the integrity of our analysis and avoid potential bias, we refrained from imputing these values. Cases with no reported ethnicity were designated as missing and were reported descriptively to provide a transparent overview of the cohort’s demographic distribution. Given the substantial proportion of missing data, the ethnicity variable was excluded from further statistical analysis. For other missing data, such as gravidity, parity, and prior preterm births, each with less than 0.6% missingness, we employed *k*-nearest neighbors (KNN) imputation. To determine the number of neighbors for imputation, we utilized a separate data set and found that using *k* = 10 nearest neighbors provided the best balance between imputation accuracy and bias.

Data were described as number and/or percentage or mean (SD), as appropriate. *P* < .05 was considered significant for all analyses and tests were 2-sided. All statistical analyses were performed using R statistical software version 4.1.3 (R Project for Statistical Computing).

## Results

### Return Rate for Oocyte Warming

A total of 77 319 autologous oocyte cryopreservation cycles were performed in the US between 2014 and 2020, as reported by the participating clinics. After excluding various medical and ART indications, the final cohort consisted of 47 363 patients undergoing planned OC cycles (Figure 1). Among these patients, 6421 (13.5%) were categorized as POR with fewer than 5 oocytes across all cycles (mean [SD] age, 36.8 [4.0] years), while the remaining 40 942 patients had 5 or more oocytes cryopreserved (mean [SD] age, 34.1 [4.7] years) (Table 1; eTable in Supplement 1). The POR group had a mean (SD) 2.8 (1.0) total vitrified oocytes, while the normal responders group had a significantly higher mean of 14.1 (8.0) oocytes (*P* < .001).

**Table 1.** Comparison of Normal and Poor Responders Undergoing Oocyte Freezing

Characteristics	Patients undergoing planned OC, No. (%)		*P* value
	Normal responders (n = 40 942)	Poor responders (n = 6421)	
Age at freezing	34.1 (4.7)	36.8 (4.0)	<.001
Return to utilize rate	1133 (2.5)	70 (3.1)	.09
Vitrification-warm interval, mean (SD), d	803.8 (160.7)	712.6 (156.1)	.27
Clinic US region			
Midwest	3821 (9.3)	736 (11.5)	<.001
Northeast	14 397 (35.2)	2699 (42.0)	
South	8939 (21.8)	1136 (17.7)	
West	13 785 (33.7)	1850 (28.8)	
Partner identity known in OC	5117 (12.5)	955 (14.9)	.12
Race and ethnicity^a^			
American Indian or Alaskan Native	177 (0.4)	16 (0.2)	.03
Asian	4816 (11.8)	827 (12.9)	.01
Black or African American	1533 (3.7)	283 (4.4)	.01
Hispanic	1282 (3.1)	206 (3.1)	.07
Native Hawaiian or Other Pacific Islander	56 (0.1)	9 (0.1)	.95
White	16 468 (40.2)	2475 (38.5)	0.01
BMI, mean (SD)	23.9 (4.3)	23.8 (4.4)	.08
Gravidity, mean (SD)	0.259 (0.7)	0.336 (0.8)	<.001
Full term births, mean (SD)	0.085 (0.3)	0.064 (0.3)	.02
Endometriosis	75 (0.2)	42 (0.7)	<.001
Polycystic ovaries	42 (0.1)	8 (0.1)	.65
Diminished ovarian reserve	468 (1.1)	400 (6.2)	<.001
Tubal ligation	1 (<0.1)	0	.82
Total retrieved, mean (SD)	18.301 (10.4)	4.867 (3.5)	<.001
Total oocyte vitrified, mean (SD)	14.166 (8.0)	2.876 (1.0)	<.001

Abbreviations: BMI, body mass index (calculated as weight in kilograms divided by height in meters squared); OC, oocyte cryopreservation.

^a^ There are 19 215 missing values in race, which constitutes approximately 40.6% of the total number of participants for race. Given the substantial proportion of missing data, the race and ethnicity variable was excluded from further statistical analysis.

In our designated planned OC cohort, a total of 1203 patients returned for oocyte warming. The rate of return for oocyte warming differed between the groups, with 260 (4.0%) in the POR group and 943 (2.3%) in the normal response group (*P* < .001).

**Figure 2.**
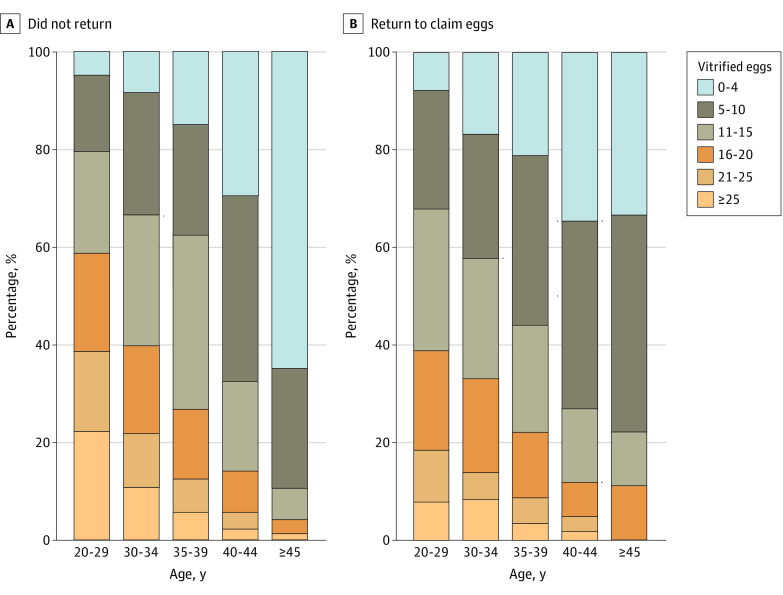
Age Stratification of Patients at the Time of Planned Oocyte Cryopreservation by Total Number of Vitrified Oocytes

Figure 2 displays the age stratification of patients at the time of planned OC, categorized by the total number of oocytes cryopreserved. The figure shows a higher rate of POR among patients who returned to utilize their eggs. Table 2 presents the rates of patients returning for oocyte utilization, comparing age subgroups and the presence of a POR diagnosis during ovarian stimulation. Diagnosis of POR at the time of ovarian stimulation was more prevalent among those who returned to utilize their oocytes. This trend was particularly evident in the 2 most common age groups for planned oocyte cryopreservation: ages 30 to 34 years and 35 to 39 years, with rates of 8.4% (982 of 11 743 patients) vs 16.3% (46 of 275 patients) (*P* < .001) and 14.9% (3433 of 23 012 patients) vs 21.1% (124 of 587 patients) (*P* < .001), respectively. eFigure 1 in Supplement 1 depicts the actual return percentages stratified by age and ovarian response, showing higher rates of return in these 2 age groups.

**Table 2.** Oocyte Freeze Numbers by Group and Age in No Warm Cycle and Warm Cycle Groups

Group	Total oocytes, range	Total patients, No. (%)	Age at freezing, patients, No. (%)				
			<30 y	30-34 y	35-39 y	40-44 y	≥45 y
No warm cycle, No.	NA	46 160)	6801	11 743	23 012	4433	171
Poor ovarian response	0-4	6161 (13.3)	327 (4.8)	982 (8.4)	3433 (14.9)	1308 (29.5)	111 (64.9)
Normal response	5-10	13 379 (29.0)	1068 (15.7)	2950 (25.1)	7629 (33.2)	1690 (38.1)	42 (24.6)
	11-15	11 202 (24.3)	1419 (20.9)	3152 (26.8)	5804 (25.2)	816 (18.4)	11 (6.4)
	16-20	7137 (15.5)	1364 (20.1)	2119 (18.0)	3277 (14.2)	372 (8.4)	5 (2.9)
	21-25	4121 (8.9)	1113 (16.4)	1284 (10.9)	1572 (6.8)	152 (3.4)	0
	>25	4160 (9.0)	1510 (22.2)	1256 (10.7)	1297 (5.6)	95 (2.1)	2 (1.2)
Warm cycle, No.	NA	1203	103	275	587	229	9
Poor ovarian response	0-4	260 (21.6)	8 (7.8)	46 (16.7)	124 (21.1)	79 (34.5)	3 (33.3)
Normal response	5-10	393 (32.7)	25 (24.3)	70 (25.5)	205 (34.9)	89 (38.9)	4 (44.4)
	11-15	261 (21.7)	30 (29.1)	68 (24.7)	128 (21.8)	34 (14.8)	1 (11.1)
	16-20	170 (14.1)	21 (20.4)	53 (19.3)	79 (13.5)	16 (7.0)	1 (11.1)
	21-25	64 (5.3)	11 (10.7)	15 (5.5)	31 (5.3)	7 (3.1)	0
	>25	55 (4.6)	8 (7.8)	23 (8.4)	20 (3.4)	4 (1.7)	0

Abbreviation: NA, not applicable.

As age and response to stimulation are not the only potential confounders that may affect rates of return, we fitted a logistic model adjusted for some of these confounders to identify patients more likely to return and utilize their oocytes (Table 3). The model identified that having fewer than 5 oocytes cryopreserved was associated with higher odds of utilizing oocytes (5 oocytes or more: odds ratio [OR], 0.97; 95% CI, 0.96-0.98) and that the Northeast (OR, 0.58; 95% CI, 0.48-0.71) and West (OR, 0.65; 95% CI, 0.53-0.80) clinic regions had lower odds of utilizing oocytes,

**Table 3.** Logistic Regression Analysis of Factors Associated With Returning to Utilizing Oocytes

**Characteristic**	Patients returning to claim oocytes, OR (95% CI)^a^
Total cryopreserved	0.97 (0.96-0.98)
Age at freezing	1.07 (1.05-1.08)
Clinic region	
Midwest	1.00 [Reference]
Northeast	0.58 (0.48-0.71)
South	1.22 (1-1.48)
West	0.65 (0.53-0.80)
BMI	1.02 (1.01-1.03)
Endometriosis	1.75 (0.68-3.68)

Abbreviations: BMI, body mass index (calculated as weight in kilograms divided by height in meters squared); OR, odds ratio.

^a^ Adjusted for confounders.

### Time to Return

We estimated the time elapsed from oocyte cryopreservation to warming (time to return) in cases where patients returned to utilize their oocytes in planned OC cycles. The overall time elapsed from cryopreservation to warm was not significantly different between the 2 groups, with a mean (SD) of 716.1 (156.1) days in the POR group and 803.8 (160.7) days in the normal response group (Table 1). The mean and median values are presented in eFigure 2 in Supplement 1. eFigure 3 in Supplement 1 presents the results of a Cox proportional hazards model analysis, which examined the effect of the response class (POR or normal responders) on the cryopreservation warm interval.

## Discussion

In our study, we observed that patients diagnosed with POR were more likely to return and utilize their cryopreserved oocytes after planned OC. Our hypothesis posited that a newly diagnosed POR, even without prior infertility history, might serve as a stressor prompting earlier attempts at conception, both unassisted and with ART, compared with those exhibiting a normal ovarian response. Recognizing that a POR in planned OC cycles poses a challenge for clinicians, we emphasize the genuine need for comprehensive counseling for these patients. By filling this research void, we aim to facilitate the design of effective strategies to aid individuals experiencing POR in this scenario.

### Study Outcomes

In this cohort study, we found that only 2.5% to 3.0% of patients returned to utilize their cryopreserved oocytes after planned OC, a rate significantly lower than that reported in previous studies.^9,15^ A notable trend observed was that patients identified as poor ovarian responders consistently demonstrated a higher likelihood of returning to utilize cryopreserved oocytes across all age groups, particularly within the common planned OC age groups of 30 to 34 years and 35 to 39 years. This pattern implies a potential influence of POR on patients’ decisions to utilize their stored oocytes. Despite low absolute numbers, women of advanced age (ie, older than 40 years) showed a decreased likelihood of coming back for utilization. This could be related to the patients already being primed to expect lower numbers of oocytes due to age and not necessarily related to a POR.

Despite the elevated rate of oocyte utilization in the POR cohort, we found no significant disparity in the time taken for either the patients with POR or normal responders to return for oocyte utilization. This observation suggests that ovarian response does not considerably impact the timing of utilization. Through logistic regression analysis, we established a statistically significant association between the total count of cryopreserved oocytes and the future likelihood of oocyte utilization. We also discovered significant effects of both BMI and clinic region within the US on the decision to utilize cryopreserved oocytes.

These findings highlight the low overall rate of patients returning to utilize cryopreserved oocytes after planned oocyte cryopreservation, the association of POR with utilization rates, and the potential role of age in predicting the timing of utilization. These results provide insights for clinicians counseling patients undergoing planned OC and contribute to a better understanding of the factors influencing oocyte utilization in this context.

### Complex Counseling Challenges and Utilization Patterns in Planned Oocyte Cryopreservation Cycles

The presence of POR in planned OC cycles presents a complex counseling challenge.^17,18^ Research suggests a potential correlation between POR and reduced likelihood of success in ART cycles. However, studies on the relationship between POR and infertility vary in their indications and may not fully represent the general population seeking planned oocyte freezing cycles.^12,13,19^ Furthermore, low ovarian reserve does not predict difficulty in conceiving spontaneously or a longer time to conceive.

In this study, the reasons and indications for patients returning to utilize their cryopreserved oocytes for pregnancy were not fully evaluated. Factors such as age, lack of a partner, or inability to conceive spontaneously may contribute to the decision to warm and use the cryopreserved oocytes with or without donor sperm. It is important to note that fertility outcomes are influenced by multiple factors, and further research is needed to better understand the relationship between ovarian reserve and fertility.^19^

The utilization rate of cryopreserved oocytes after oocyte preservation varies across studies and patient populations. Similar to other studies, our findings indicate that currently, only 2.5% to 3.0% of patients have returned to utilizing their oocytes. Cobo et al^5^ reported a utilization rate of 9.3% in a retrospective study, while a more recent multicenter study^15^ by the same research group observed an increased utilization rate of 12.1%. It is important to note that the utilization rate can be influenced by factors such as the patient population, length of follow-up, and potential confounding by indication. In our previous single-center analysis,^20^ we found that 7.4% of patients returned to warm in our practice.

Additionally, there is no consensus on how POR affects success in ART cycles, complicating counseling for patients with this condition.^21^ The unpredictability in the patterns of returning to use stored oocytes, especially among those with POR, adds to the counseling complexity. Furthermore, the ethical and logistical issues arising from the large number of unused cryopreserved oocytes pose significant dilemmas. Lastly, decisions to utilize stored oocytes are influenced by a multitude of factors, ranging from biological aspects like age to personal situations such as the absence of a partner, further complicating the decision-making process. These variations highlight the challenges associated with oocyte utilization and emphasize the need for further research in this area.

This study also found that the time to return to utilize their cryopreserved oocytes was not significantly different between the POR group and the normal responder group. This suggests that factors other than the number of oocytes or ovarian response, such as personal circumstances, treatment plans, or individual preferences, may primarily influence the decision-making process and timing of utilization.

The findings highlight the need for ongoing counseling and discussions with patients undergoing planned oocyte freezing to address the complexities of POR, the limited return rates, and the potential challenges associated with the increasing number of unused cryopreserved oocytes. Further research is warranted to explore these issues and optimize the decision-making process for patients considering oocyte freezing for future fertility.

The time frame for the return of cryopreserved oocytes can vary among studies, and it is influenced by several factors, including the age at which the oocytes were vitrified, the duration of storage, and the intention of using the frozen oocytes. While not all studies have provided specific time frames for the return rates, some findings suggest conflicting implications regarding the utilization of cryopreserved oocytes based on the age at vitrification. Similar to our results, which showed a constant and gradual increase to utilize with age, Leung et al^20^ observed that individuals who eventually utilized their frozen eggs tended to be from older age groups (38 years or older), suggesting a possible preference among women of advanced age to return for thawing and using their cryopreserved oocytes. However, as absolute numbers are small, more research is needed to capture the factors influencing the timing and rates of returning for oocyte thawing and utilization, considering various aspects of fertility preservation and assisted reproductive technologies.

In light of these considerations, we make the following recommendations. For clinicians, given the increased likelihood of patients with POR returning for oocyte warming, a proactive approach in counseling is recommended. This should include discussing the clinical, emotional, and ethical aspects related to the use of cryopreserved oocytes, and the challenges that may arise for patients. For patients with POR, we recommend they should be fully informed about the implications of their condition, including the potential need for additional oocyte preservation cycles and the availability of supportive resources to assist with the complexities of decision-making. And for researchers, we emphasize the need for a deeper understanding of why certain patients are more likely to utilize their cryopreserved oocytes is clear. Future studies should focus on elucidating these motivations,

### Strengths and Limitations

We focused on several clinically relevant outcomes that are imperative in counseling future planned OC patients, including the rate of patients who returned for oocyte warming, the time interval between cryopreservation and oocyte retrieval, and the live birth rate among patients who reutilized their cryopreserved oocytes. This study benefits from a national quality-controlled reporting system and database, allowing for a diverse and representative sample. It examines multiple clinically relevant outcomes, including the rate of patients returning for oocyte warming and the timing of utilization. By addressing a research gap in understanding the factors influencing oocyte utilization in planned OC, the study may assist clinicians and researchers. Additionally, it compares the utilization rates with existing research, highlighting the variability in rates reported.

However, our study does have several limitations that should be acknowledged. First, we were unable to fully categorize autologous OC cycles based on precise indications for treatment. This limitation arose due to missing data on the indication for oocyte cryopreservation, necessitating the use of inferred indications based on comorbidities, cycle designation, and free-text descriptions. Consequently, there may be some degree of misclassification in the categorization of cycles, which could potentially impact the labeling of the specific indications. Although the study encompassed data from multiple clinics on a national scale, it is important to note that detailed information on individual cycles was not accessible, which could potentially affect the generalizability of our findings and hinder the development of more accurate models to simulate return time intervals.

Additionally, our study lacks information regarding the indication for warming and future unassisted pregnancies, thereby preventing us from providing insights into the reasons why patients did or did not return for oocyte warming. This information could have offered insights into patient decision-making and informed consent.

An inherent limitation of our study is the absence of data on prior unassisted conception attempts among participants before undergoing planned oocyte cryopreservation (OC). This gap hinders our understanding of their full reproductive history and may affect the interpretation of OC utilization rates. Future studies should consider this variable to enrich OC counseling and decision-making frameworks.

We acknowledge the limitation of our data set in providing a granular understanding of the multifaceted personal circumstances that influence the decision-making process. While our analysis attempts to adjust for some of these through known variables, deeper qualitative studies might be required to holistically appreciate the individual nuances that guide patients in their decision to utilize cryopreserved oocytes.

Despite efforts to match oocyte cryopreservation and thaw cycles using SART data, we recognize the limitations of potential missing or unlinked data. Such gaps could bias outcome assessments. While our linkage rate was high, these database limitations may affect the interpretation of oocyte utilization rates.

Lastly, we opted to exclude cycles in which individuals returned to utilize their oocytes within 60 days after oocyte cryopreservation. Although this time frame may be relatively short for typical patients undergoing planned OC, we made this exclusion to ensure we captured those individuals who returned to utilize cryopreserved oocytes immediately following poor oocyte yield. Future research addressing these limitations could provide further insights into the specific indications, patient decision-making, and factors influencing return rates and outcomes in oocyte cryopreservation cycles.

## Conclusions

Drawing from the data of this study, there is a need for nuanced counseling that caters to the unique ovarian response of each patient. For patients with poor response, there is a discernibly higher propensity to utilize cryopreserved oocytes. Such patients should be informed of this trend and its potential implications on their fertility journey. For those with a normal ovarian response, despite the observed lower utilization rates, the decision to freeze more or fewer oocytes should not be predominantly influenced by these rates alone. Other pivotal considerations include individual circumstances, long-term reproductive goals, and potential future shifts in utilization patterns. An important observation to highlight is the common practice among many clinicians to pivot from OC to ART or IVF in instances of POR. This suggests that the return rates in patients with POR might actually be more prevalent than our data indicate, as some patients with very poor responses might have already shifted to ART or IVF in a shorter time frame than we were able to investigate.

Given the heightened likelihood of poor responders using their cryopreserved oocytes, one may advocate for poor responders to undergo additional stimulation cycles to enhance their oocyte reserve, in line with the practical outcomes presented in prior studies.^3,20^ This strategy could potentially lead to improved success rates for these patients. Comprehensive counseling should ensure that patients are well-versed with the distinct utilization patterns across different ovarian response groups. Equipped with this knowledge, they can make decisions in line with their reproductive goals and prospective scenarios. Ultimately, this study accentuates the paramount importance of tailored counseling, blending empirical evidence with the unique attributes and inclinations of each patient.

## Notes

Supplement 1. **eTable.** Study Cohort Overview **eFigure 1.** Actual Return Percentages Stratified by Age and Ovarian Response **eFigure 2.** Time Elapsed From Vitrification to Warm in Patients Undergoing POC (Oocyte Cryopreservation) Who Returned to Utilize Their Oocytes **eFigure 3.** Cox Proportional Hazards Model Analysis for Vitrification Warm Interval

Supplement 2.
Data Sharing Statement
