# Supplementary material for: Utilization of Cryopreserved Oocytes in Patients With Poor Ovarian Response After Planned Oocyte Cryopreservation
Source: JAMA Netw Open. 2024 Jan 2;7(1):e2349722. doi: 10.1001/jamanetworkopen.2023.49722 (PMC10762568; doi:10.1001/jamanetworkopen.2023.49722)
Supplement: Supplement 1. — eTable. Study Cohort Overview eFigure 1. Actual Return Percentages Stratified by Age and Ovarian Response eFigure 2. Time Elapsed From Vitrification to Warm in Patients Undergoing POC (Oocyte Cryopreservation) Who Returned to Utilize Their Oocytes eFigure 3. Cox Proportional Hazards Model Analysis for Vitrification Warm Interval [file jamanetwopen-e2349722-s001.pdf]

Supplemental Online Content

Fouks Y, Sakkas D, Bortoletto PE, Penzias AS, Seidler EA, Vaughan DA. Utilization of cryopreserved oocytes in patients with poor ovarian response, after planned oocyte cryopreservation. *JAMA Netw Open.* 2024;7(1):e2349722. doi:10.1001/jamanetworkopen.2023.49722

eTable. Study Cohort Overview

eFigure 1. Actual Return Percentages Stratified by Age and Ovarian Response

eFigure 2. Time Elapsed From Vitrification to Warm in Patients Undergoing POC (Oocyte Cryopreservation) Who Returned to Utilize Their Oocytes

eFigure 3. Cox Proportional Hazards Model Analysis for Vitrification Warm Interval

This supplemental material has been provided by the authors to give readers additional information about their work.

© 2023 Fouks Y et al. *JAMA Network Open*.

eTable. Study Cohort Overview

	Poor ovarian response	Normal ovarian response						p value
**Oocyte group**	0-4 (N=6421)	5-10 (N=13772)	11-15 (N=11463)	16-20 (N=7307)	21-25 (N=4185)	25+ (N=4215)	Total (N=47363)	p value
**Age**	36.869 (4.095)	35.657 (4.097)	34.476 (4.358)	33.510 (4.746)	32.445 (5.008)	31.300 (5.249)	34.533 (4.741)	< 0.001
**Came_back to warm rate**	260 (4.0%)	393 (2.9%)	261 (2.3%)	170 (2.3%)	64 (1.5%)	55 (1.3%)	1203 (2.5%)	< 0.001
**Time to return**	712.6 (575.0)	827.3 (585.8)	843.5 (593.0)	804.4 (650.2)	894.6 (644.4)	657.9 (623.9)		0.07*
**Clinic Region USA**								< 0.001
Midwest	736 (11.5%)	1355 (9.8%)	1072 (9.4%)	679 (9.3%)	377 (9.0%)	338 (8.0%)	4557 (9.6%)	
Northeast	2699 (42.0%)	5327 (38.7%)	4231 (36.9%)	2447 (33.5%)	1256 (30.0%)	1136 (27.0%)	17096 (36.1%)	
South	1136 (17.7%)	2942 (21.4%)	2420 (21.1%)	1599 (21.9%)	948 (22.7%)	1030 (24.4%)	10075 (21.3%)	
West	1850 (28.8%)	4148 (30.1%)	3740 (32.6%)	2582 (35.3%)	1604 (38.3%)	1711 (40.6%)	15635 (33.0%)	
**Partner Identity Known**	955 (14.9%)	1944 (14.1%)	1490 (13.0%)	874 (12.0%)	430 (10.3%)	379 (9.0%)	6072 (12.8%)	< 0.001
**Ethnicity**								
American Indian\Alaska Native	16 (0.2%)	49 (0.4%)	42 (0.4%)	37 (0.5%)	27 (0.6%)	22 (0.5%)	193 (0.4%)	0.014
Asian	827 (12.9%)	1695 (12.3%)	1487 (13.0%)	829 (11.3%)	415 (9.9%)	390 (9.3%)	5643 (11.9%)	< 0.001
Black African American	283 (4.4%)	493 (3.6%)	373 (3.3%)	283 (3.9%)	170 (4.1%)	214 (5.1%)	1816 (3.8%)	< 0.001
**BMI**	23.835 (4.4)	23.953 (4.4)	23.971 (4.3)	23.855 (4.2)	23.785 (4.6)	23.665 (3.7)	23.886 (4.3)	< 0.001
**Endometriosis**	42 (0.7%)	39 (0.3%)	17 (0.1%)	14 (0.2%)	2 (0.0%)	3 (0.1%)	117 (0.2%)	< 0.001
**PolycysticOvaries**	8 (0.1%)	12 (0.1%)	9 (0.1%)	10 (0.1%)	4 (0.1%)	7 (0.2%)	50 (0.1%)	0.602
**Diminished Ovarian Reserve**	400 (6.2%)	302 (2.2%)	106 (0.9%)	39 (0.5%)	14 (0.3%)	7 (0.2%)	868 (1.8%)	< 0.001
**Tubal Ligation**	0 (0.0%)	0 (0.0%)	0 (0.0%)	0 (0.0%)	0 (0.0%)	1 (0.0%)	1 (0.0%)	0.069
**Uterine**	16 (0.2%)	19 (0.1%)	21 (0.2%)	13 (0.2%)	7 (0.2%)	5 (0.1%)	81 (0.2%)	0.552
**Unexplained**	0 (0.0%)	0 (0.0%)	0 (0.0%)	0 (0.0%)	0 (0.0%)	0 (0.0%)	0 (0.0%)	
**Total oocyte vitrified**	2.876 (1.0)	6.989 (1.3)	11.808 (1.4)	16.782 (1.3)	21.767 (1.4)	31.944 (7.1)	12.635 (8.4)	< 0.001
**Total Retrieved**	4.867 (3.5)	9.870 (4.1)	15.593 (4.5)	21.426 (5.1)	27.283 (5.7)	38.879 (10.2)	16.480 (10.8)	< 0.001

* Analysis of Variance p value

© 2023 Fouks Y et al. *JAMA Network Open*.

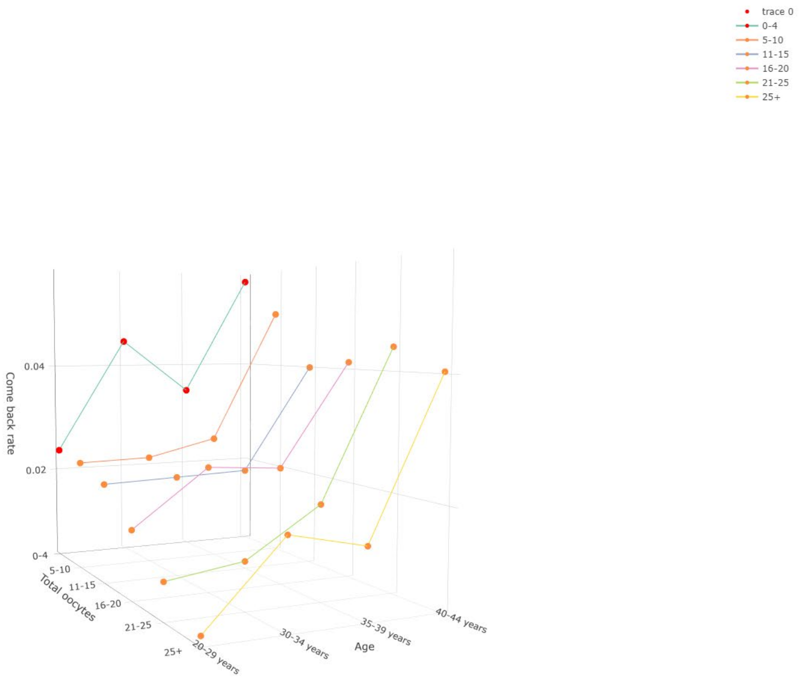

eFigure 1: Actual Return Percentages Stratified by Age and Ovarian Response 535

This figure provides the actual return percentages stratified by age and ovarian response.

© 2023 Fouks Y et al. *JAMA Network Open*.

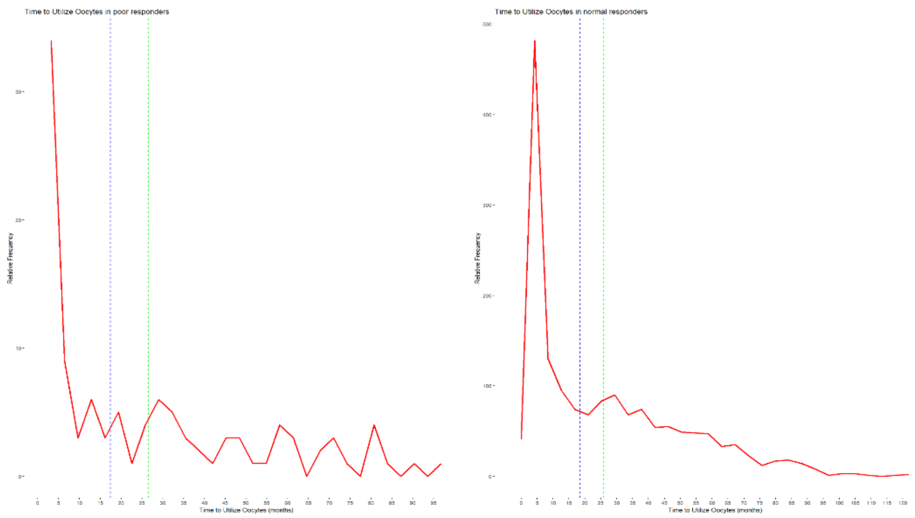

eFigure 2: Time elapsed from vitrification to warm in patients undergoing POC (Oocyte Cryopreservation) who returned to utilize their oocytes. The mean and median time elapsed are presented.

© 2023 Fouks Y et al. *JAMA Network Open*.

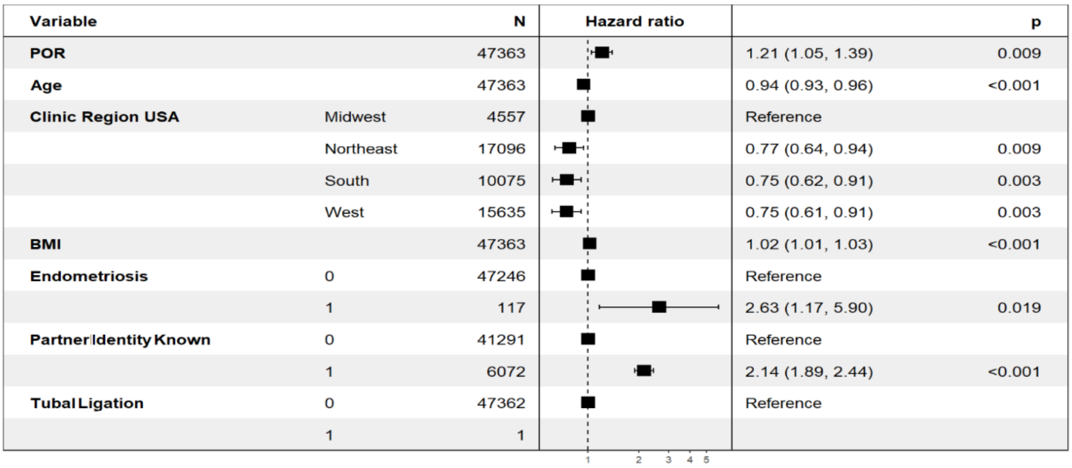

Variable		N	Hazard ratio		p
**POR**		47363		1.21 (1.05, 1.39)	0.009
**Age**		47363		0.94 (0.93, 0.96)	<0.001
**Clinic Region USA**	Midwest	4557		Reference	
	Northeast	17096		0.77 (0.64, 0.94)	0.009
	South	10075		0.75 (0.62, 0.91)	0.003
	West	15635		0.75 (0.61, 0.91)	0.003
**BMI**		47363		1.02 (1.01, 1.03)	<0.001
**Endometriosis**	0	47246		Reference	
	1	117		2.63 (1.17, 5.90)	0.019
**Partner Identity Known**	0	41291		Reference	
	1	6072		2.14 (1.89, 2.44)	<0.001
**Tubal Ligation**	0	47362		Reference	
	1	1			

eFigure 3: Cox Proportional Hazards Model Analysis for Vitrification Warm Interval

eFigure 3 presents the results of a Cox Proportional Hazards Model analysis, modeling the effect of the response class (poor ovarian response or normal responders) on the vitrification warm interval. It demonstrates the hazard ratios (HR) and significance levels for the variables included in the model, such as age, clinic region, pretreatment diagnosis of endometriosis, and partner at the time of oocyte cryopreservation.

© 2023 Fouks Y et al. *JAMA Network Open*.
