# Supplementary material for: Utilization of Cryopreserved Oocytes in Patients With Poor Ovarian Response After Planned Oocyte Cryopreservation
Source: JAMA Netw Open. 2024 Jan 2;7(1):e2349722. doi: 10.1001/jamanetworkopen.2023.49722 (PMC10762568; doi:10.1001/jamanetworkopen.2023.49722)
Supplement: Supplement 2. — Data Sharing Statement [file jamanetwopen-e2349722-s002.pdf]

Data Sharing Statement

Fouks. Utilization of Cryopreserved Oocytes in Patients With Poor Ovarian Response After Planned Oocyte Cryopreservation. *JAMA Netw Open*. Published January 02, 2024. doi:10.1001/jamanetworkopen.2023.49722

Data
Data available: Yes
Data types: Other (please specify)
Additional Information: The use of data needs to be aproved by the SART CORS
How to access data: https://www.sart.org/professionals-and-providers/research/
When available: With publication

Supporting Documents
Document types: None

Additional Information
Who can access the data: anyone requesting the data
Types of analyses: for any purpose
Mechanisms of data availability: SART approval
